# Th1/Th2 Dichotomy in Obese Women with Gestational Diabetes and Their Macrosomic Babies

**DOI:** 10.1155/2018/8474617

**Published:** 2018-11-14

**Authors:** A. Seck, A. Hichami, S. Doucouré, F. Diallo Agne, H. Bassène, A. Ba, C. Sokhna, N. A. Khan, A. Samb

**Affiliations:** ^1^Laboratory of Physiology and Functional Explorations, Faculty of Medicine, Pharmacy and Odontology, Cheikh Anta Diop University, 5005 Dakar-Fann, Senegal; ^2^U1231 INSERM/Université de Bourgogne-Franche Comté (UBFC)/Agro-Sup, Physiologie de la Nutrition & Toxicologie, Dijon 21000, France; ^3^Institute of Research for Development, VITROME Aix Marseille Univ, IRD, AP-HM, SSA, VITROME, IHU-Mediterranean Infection, CP18524 Dakar, Senegal; ^4^Laboratory of Biochemistry and Molecular Biology, Faculty of Medicine, Pharmacy and Odontology, Cheikh Anta Diop University, 5005 Dakar-Fann, Senegal; ^5^UMI 3189, “Environnement, Santé, Sociétés”, CNRS, CNRST, Université Bamako-UCAD, Dakar, Senegal

## Abstract

The aim of the study was to assess T cell differentiation and the modulation of inflammatory cytokines in obese and gestational diabetes mellitus (GDM) women and their macrosomic newborns. Hence, immediately after delivery, blood samples were collected through the mother's arm vein and the umbilical cordon vein. Biochemical parameters measured were HbA1C, glucose, insulin, triglyceride (TG), total cholesterol (Tchol), HDL cholesterol (HDLchol), and LDL cholesterol (LDLchol). T lymphocytes were purified from the total blood with Ficoll-Paque. The mRNA expression of inflammatory markers in T cells was determined by RT-qPCR. We observed that diabetic mothers exhibited higher HbA1C, glycemia, insulinemia, TG, Tchol, HDLchol, and LDLchol levels than control mothers. Glycemia was not significantly different between macrosomic and control newborns. However, insulinemia was high in macrosomic babies. TG, Tchol, HDLchol, and LDLchol were not significantly different between macrosomic and control babies. In diabetic mothers, mRNA expression of the Th1 cell subtype was significantly increased. Th1 markers were upregulated in babies born to diabetic women than in control newborns. However, expression of two Th2 markers (GATA3 and IL-4) was not significantly different between control and GDM women and between their respective newborns. Interestingly, IL-10 mRNA expression was decreased in diabetic mothers and their offsprings. The Th1/Th2 cytokine ratio was increased in GDM obese mothers and their macrosomic newborns, suggesting a proinflammatory status in these subjects.

## 1. Introduction

Gestational diabetes mellitus (GDM) is defined as a state of glucose intolerance during pregnancy [1]. A sedentary and the modern lifestyle in developing countries contributes to the increased prevalence of the GDM. In studies, the global prevalence of GDM was estimated to be 1%–14% according to the population studied [2–5]. Gestational diabetes mellitus is also associated with increased risk for a mother and her offspring in the short and long term. About 50% of GDM women develop type II diabetes in 5–10 years after their pregnancy [4]. Gestational diabetes is responsible for perinatal complexities such as fetal malformations, growth anomalies, and miscarriages induced by high blood pressure and preeclampsia [6]. Several studies support the hypothesis that maternal hyperglycemia in the second half of pregnancy may lead to an increased fetal weight. Indeed, some authors have reported the relationship between gestational diabetes and fetal macrosomia [7, 8]. Macrosomia is observed in 50% of pregnancies with gestational diabetes, being one of the main reasons of increased perinatal morbidity and mortality [8, 9]. In the long term, children born to gestational diabetic women present a high risk to develop obesity and type II diabetes in adolescence [2]. It has been clearly established that both obese and type II diabetic subjects suffer from having a proinflammatory status [10, 11]. Adipose tissue of obese people has been shown to release several cytokines such as IL-6 and TNF-*α* favouring inflammation and insulin resistance [12, 13].

The immune system is composed of two major subdivisions, the innate immune system and the adaptive immune system. The innate immune cells control the adaptive immune response through the activation and induction of differentiation of naive T helper (Th0) cells to Th1, Th2, Treg, or Th17 effector cells. Th1 cells, by the production of IL-2 and IFN-*γ*, trigger a cell-mediated immunity response through the activation of macrophages and cytotoxic T cells, whereas Th2 cells trigger a humoral immune response by the production IL-4, IL-5, and IL-13 which activate IgE antibody-producing B cells [13]. Thus, it has also been shown that the immune system, particularly T helper lymphocytes (Th cells), by their capacity to differentiate into either pro- or anti-inflammatory Th cells, plays a pivotal role during the development of insulin resistance [13].

Differentiation of Th cells into Th1 and Th2 cells involves the activation of Th1 and Th2 transcription factors, T-bet and GATA3, respectively [14, 15]. It has been reported that abnormalities of humoral and cell-mediated immunity in type I diabetic women during pregnancy may complicate immune-fetal interaction leading to fetal overgrowth and alteration in the immune system of a newborn which persist till adulthood [16, 17].

With regard to gestational diabetes, several reports have analysed the expression of cytokines by ELISA in serum or by RT-PCR in the pancreas and spleen of gestational diabetes rats [18] and mice [19]. A few studies have reported in nonobese human GDM the production of proinflammatory cytokines measured either by ELISA in serum or by RT-PCR in the placenta [19–21]. The originality of our study is that we selected only obese GDM mothers and their macrosomic offsprings and we determined Th1/Th2 lymphocyte differentiation by measuring their respective mRNA in purified T cell lymphocytes from these subjects.

## 2. Subjects and Methods

### 2.1. Subjects and Protocol

#### 2.1.1. Subjects

This study was conducted at the gynecology department of Roi Baudouin Guediawaye Hospital, Dakar Principal Hospital, and Health Center Dakar Plateau between July 2016 and April 2017. The study protocol was approved by the National Ethical Committee of Senegal. All subjects were informed about the procedure and purpose of the study. The written informed consent was obtained from all the participants.

We recruited 20 pregnant women and their newborns (*n* = 20). Women aged from 24 to 39 years were between their 20th and 30th week of gestational period. These subjects were divided into two groups; (i) the first group (G1), considered the control group, included normoglycemic pregnant subjects without any history of illness or risk factors for gestational diabetes and (ii) the second group (G2) included women with gestational diabetic mellitus (GDM) diagnosed during pregnancy by the oral glucose tolerance test (OGTT) and they had not received insulin treatment. All pregnant women included in this study had no history of smoking and were not taking decoction or medicine, which could disturb the pregnancy evolution.

Immediately after delivery, blood samples were collected through the mother's arm vein and the umbilical cord vein.

#### 2.1.2. Experimental Protocol

*(1) Blood Samples*. After delivery, fasting venous samples were collected in either EDTA or dry tubes (without anticoagulant). The blood collected in the EDTA tube was used for determination of the HbA1C (glycated hemoglobin) level. Blood samples in the dry tube or in EDTA were centrifuged to obtain, respectively, serum and plasma. Samples were aliquoted and frozen at −80°C for determination of other biochemical parameters.

*(2) Determination of Biochemical Parameters*. Biochemical parameters were analysed by using an automatic spectrometer (chemistry module LEc4000 of architect system ci4100). We determined plasma glucose concentrations and serum levels of insulinemia, triglyceride (TG), total cholesterol (Tchol), and high-density lipoprotein cholesterol (HDLchol). Low-density lipoprotein cholesterol (LDLchol) concentrations were calculated by the Friedewald formula: LDLchol = Tchol–HDLchol–TG/5.

*(3) T Cell Isolation*. PBMC were isolated from the blood with EDTA using Ficoll-Paque™. T lymphocytes were isolated from PBMC by panning as described elsewhere [22]. Briefly, PBMC were washed once with PBS-containing bovine serum albumin (2 g/L) and seeded in a Petri dish. After 2 h, nonadherent cells were gently decanted and transferred to antihuman IgG-coated Petri dishes. This allowed adhesion of B lymphocytes to the substratum of the Petri dishes. After one hour of incubation, T lymphocyte-rich supernatant was decanted and washed twice with PBS-BSA and resuspended in RPMI 1640 medium. This technique provided us with an enriched T cell population (99%), verified by flow cytofluorometry (data not shown).

*(4) Detection of mRNA Expression of Inflammatory Markers in T Cells by RT-qPCR*. Total RNA of T lymphocytes was extracted using TRIzol® Reagent, the quality of RNA was evaluated with the optical density (OD) at 260 nm–280 nm, and the RNA/DNA ratio was measured by spectrophotometry (Jenway Genova).

Real-time qPCR (RT-qPCR) was performed with StepOne software V2.3 using Power SYBR Green PCR Master Mix (Thermo Fisher Scientific) and oligonucleotide human primers (Table 1). We used *β*-actin as the reference gene since it has shown the most relative stability. The relative gene expression was determined using the ΔΔCt method. The normalized delta cycle threshold (ΔCt) was calculated by subtracting the cycle threshold (Ct) value of the genes of interest from the *β*-actin cycle threshold value (ΔCt = Ct*β* − actin–Ctgene). Comparative gene expression between two independent samples (ΔΔCt) was obtained by subtracting the delta cycle threshold of the control group from the delta cycle threshold of the group of interest (obese or macrosomic).

*(5) Statistical Analysis*. Samples were analysed using GraphPad Prism version 5.2. Data expressed as mean ± standard deviation (SD) were evaluated by one-way ANOVA. The Fisher test and the Student *t*-test were used to compare the values between two groups. Differences were considered significant when the *p* value was <0.05.

## 3. Results

### 3.1. Medical and Obstetrical Background

Regarding the medical and obstetrical background, GDM women presented more obstetrical complications than their preceding pregnancy. Recurrent spontaneous abortions were frequent in GDM mothers (70%). Miscarriage and stillbirth were ranged from 20% to 30% on gestational diabetes. Other previous complications such as preeclampsia and high blood pressure were less frequent (10%) (Table 2).

### 3.2. Anthropometrical Parameters

We observed that the control group (mothers and their newborns) had normal BMI. All of gestational diabetic women included in the study were obese, and their newborns were macrosomic with a birth weight over 4 kg. The cranial perimeter of macrosomic newborns was significantly higher than that of the control newborns (Table 3).

### 3.3. Biochemical Parameters

Diabetic mothers exhibited a higher level of fasting glycemia and insulinemia than control mothers. Compared to nondiabetic mothers, blood HbA1C percentage was increased in gestational diabetic women. Plasma glycemia was not significantly different between macrosomic and control newborns. However, the serum insulinemia level was high in the macrosomic babies.

As regards the lipid parameters, gestational diabetic women had significantly higher levels of triglycerides, total cholesterol, HDL cholesterol, and LDL cholesterol compared to nondiabetic women. Serum triglycerides, total cholesterol, HDL cholesterol, and LDL cholesterol were not significantly different between macrosomic newborns and babies born to control mothers (Table 3).

### 3.4. Expression of Pro- and Anti-Inflammatory mRNA Markers (Th1/Th2) in T Cells

**Figure 1:**
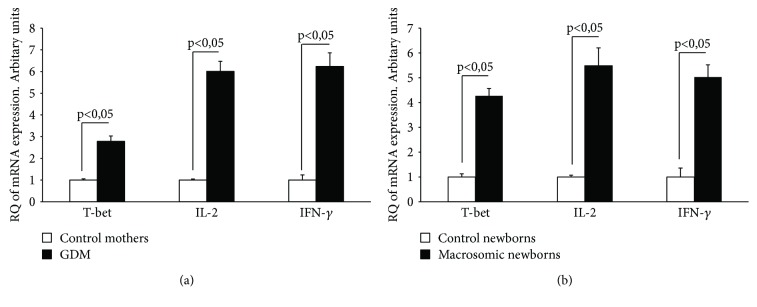
Th1 markers in T cells: mRNA expression of Th1 transcriptional factor (T-bet) and Th1 cytokines (IL-2, IFN-*γ*) in GDM and control mothers (a) and their newborns (b). Relative quantity (RQ) of mRNA in different groups was determined as described in Subjects and Methods.

**Figure 2:**
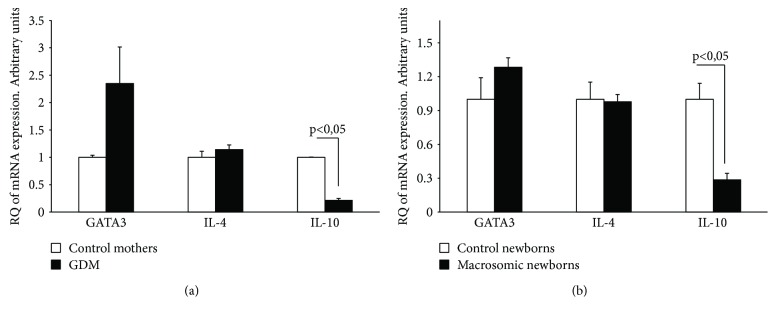
Th2 markers in T cells: mRNA expression of Th2 transcriptional factor (GATA3) and Th2 cytokines (IL-4, IL-10) in GDM and control mothers (a) and their newborns (b). Relative quantity (RQ) of mRNA in different groups was determined as described in Subjects and Methods.

In T cells of diabetic gestational mothers, the mRNA expression of Th1 transcriptional factor (T-bet) and Th1 cytokines (IL-2 and IFN-*γ*) was significantly higher compared to that in T cells of nondiabetic mothers (Figure 1(a)). Th1 markers were more expressed in babies born to gestational diabetic women than in newborns of control mothers (Figure 1(b)). Analysis of mRNA expression of Th2 transcriptional factor (GATA3) and IL-4 (Th2 cytokine) showed that there was no significant difference between control and diabetic women and between their respective newborns (Figures 2(a) and 2(b)). However, IL-10 expression (Th2 cytokine) was decreased in T lymphocytes from diabetic gestational mothers and in their offsprings (Figures 2(a) and 2(b)).

In gestational diabetic mothers and control mothers and their corresponding babies, the Th1/Th2 ratios were calculated: T-bet/GATA3, IL-2/IL-4, IL-2/IL-10, IFN-*γ*/IL-4, and IFN-*γ*/IL-10. The obtained ratio showed that the profile of Th1 transcriptional factor and Th1 cytokines was upregulated, while Th2 markers were downregulated, in T lymphocytes from both obese gestational diabetic mothers and their macrosomic newborns, compared to their respective controls (Table 4).

## 4. Discussion

Both obesity and gestational diabetes mellitus (GDM) complications during pregnancy can substantially influence the development of offspring during fetal life and postnatally. Several complications have been associated with gestational diabetes as well as with macrosomia, including metabolic abnormalities, alteration in antioxidant status, and dysregulation of the immune system [23].

This study was conducted to investigate Th1/Th2 T cell polarisation in obese mothers with GDM and their macrosomic offspring. GDM confers a risk of obstetrical complications during pregnancy [2]. The high frequency of obstetrical background observed in our gestational diabetic women corroborates the observations of several studies that have reported links between gestational diabetes and high risk of obstetrical complications during pregnancy [2, 24].

By the anthropometric view, GDM mothers of the study population were obese and their newborns were macrosomic. Biochemical data obtained in the present study showed that gestational diabetic women were hyperglycemic, hyperinsulinemic with HbA1C, and lipidic with levels higher than those of control mothers. The HbA1C elevation shows that glycemic balance was better in the control group than in the gestational diabetic one. Hyperglycemia associated with hyperinsulinemia observed in GDM mothers represents an insulin-resistant state as shown in several reports [20, 25]. Obesity constitutes a favourable situation for diabetes development. Moreover, gestational diabetes mellitus and obesity during pregnancy significantly influence the development of offspring during fetal life and postnatally. Indeed, animal and human studies indicated that fetuses from mothers with gestational diabetes are at high risk of developing fetal macrosomia and they are prone to adverse side effects strongly associated with prematurity, birth trauma, respiratory distress syndrome, and fetal death [24].

Macrosomic newborns were normoglycemic but hyperinsulinemic and had increased lipid levels compared to the control newborns. According to several studies, high glucose level of a gestational diabetic mother may induce the release of insulin from the fetal pancreas, leading to fetal hyperinsulinemia [25–27]. Besides, Grissa et al. demonstrated correlations with obesity in maternal levels of amino acids, triglycerides, fatty acids, and birth weight [28]. These substances might modulate insulin secretion and insulin sensitivity and increase fetal growth [28–30]. The hyperinsulinemia, associated with other growth factors like IGF-1 and IGF-2, could be responsible for hypoglycemic accidents of newborns [28].

Commonly, macrosomic newborns of diabetic mothers used to be hypoglycemic after birth as a consequence of their hyperinsulinemia. In our study, the hyperglycemic babies from obese diabetic pregnancies showed normoglycemia. Studies have shown that, compared to pregnancies in nonobese diabetic subjects, gestational diabetes in obese subjects is characterized by lower weight gain and higher baseline glucose [31]. This fact could explain the normoglycemic status of the newborns from obese diabetic mothers.

T cells (responsible for cell-mediated immunity) have been shown to play a critical role in GDM and macrosomia [13, 17, 18]. T cells can be divided into two subsets: T helper cells (CD4+) and T cytotoxic cells (CD8+). Prolonged stimulation of Th0 cells with a specific antigen allows their differentiation into two phenotypes: Th1 or Th2, characterized by different profiles of cytokines. Th1 cells (IFN-*γ*) inhibit the proliferation of Th2 cells, whileTh2 cells (IL-10) block the Th1 cytokine processing. T cell differentiation into Th1 and Th2 phenotypes is dependent, respectively, on T-bet and GATA3. Two transcriptional factors allow the transcriptional activation of cytokine genes [14].

In the present study, T-bet and Th1 cytokines were increased in both gestational diabetic mothers and macrosomic newborns, while, except for the downregulation of IL-10, there was no significant modulation of GATA3 and IL-4, the Th2 markers, in both diabetic women and their macrosomic offsprings. IL-10 is mainly produced by activated macrophages and Treg and Th2 cells. IL-10 inhibits the production of IFN-*γ* by Th1 cells, which shifts immune responses toward a Th2 type [15, 31].

Also, Mayer and Hudrisier [32] reported that pregnancies in obese women have a dysregulated maternal cytokine profile with a significant rise in proinflammatory cytokines [32–34]. These observations suggest the inflammatory state in obese GDM women.

In normal pregnancy, Th1 cytokines are downregulated whereas cytokines belonging to Th2 cells are upregulated at the end of the first trimester and at delivery [35]. Besides, a shift of a Th1 phenotype to Th2 during pregnancy has been shown to encourage vigorous production of antibodies that not only combat infections during pregnancy but also offer passive immunity to the fetus [36]. In this present study, the fetal passive immunity might be compromised by the decrease in the Th2 phenotype in gestational diabetic mothers.

Atègbo et al. [20] have demonstrated the downregulation of the Th1 phenotype. Contrary to that, in our obese gestational diabetes women and their macrosomic newborns, the Th1/Th2 ratio reveals the upregulation of the Th1 phenotype. Obesity of mothers might involve a Th1 increase. Our idea can be supported by observations of Khan et al. who developed an animal model of macrosomic offspring of rats which become obese and diabetic in adulthood. They observed a very high level of proinflammatory cytokines, IFNg and IL2, in obese and diabetic animals [18]. In gestational diabetic women, some authors have shown that low Th1 concentrations and high IL-10 levels may be implicated in maintaining the pregnancy [13, 17, 20]. Besides, recent data have suggested the protective role of IL-10 against inflammation in adipose tissue and insulin resistance [37–39].

Furthermore, some authors [17, 40] have reported spontaneous abortion cases and patients with recurrent miscarriage are associated with lower systemic IL-10 compared to normal pregnancies. Jasper et al. [41] have shown that adoptive transfer of T lymphocytes depleted of Treg cells in pregnant T cell-deficient mice led to a failure of gestation due to immunological rejection of the fetus [17, 41].

In our study, the increase in Th1 and decrease in IL-10 can be also explained by the status of GDM mothers with multiple background of recurrent miscarriage and spontaneous abortion.

We have not observed any significant variation in GATA3 and IL-4, which is in line with previous reports [18–20]. The lack of decrease in mRNA expression of GATA3 and IL-4 would lead to a successful pregnancy in these obese women with GDM.

## 5. Conclusion

In the context of gestational diabetes with obesity and multiple background of recurrent abortion, Th1 transcriptional factor and cytokines are upregulated while GATA3 and IL-4 (belonging to the Th2 phenotype) are not altered in diabetic mothers and their newborns. IL-10, belonging to both Treg and Th2, is decreased. The modulation of Th1/Th2 balance during pregnancy could explain the proinflammatory status observed in diabetic mothers and their offsprings.

## Figures and Tables

**Table 1:** Sequences of PCR primers.

Genes amplified	Human primer sequences
Actin	Forward 5′ATG ATA TCG CCG CGC TCG TCG TC 3′
	Reverse 5′AGG TCC CGG CCA GCC AGG TCC AG 3′

T-bet	Forward 5′TGT CCT ACT ACC GAG GCC AG 3′
	Reverse 5′ATC TCA GTC CAC ACC AAG GG 3′

IFN-*γ*	Forward 5′AGC ACT GGC TCA GAT TGC AGG C3′
	Reverse 5′ATC TCA GGG GCC AAC TAG GCA G3′

IL-2	Forward 5′CAC TAA TTC TTG CAC TTG TCA C 3′
	Reverse 5′CCT TCT TGG GCA TGT AAA ACT 3′

GATA3	Forward 5′CTC ATT AAG CCC AAG CGA AG 3′
	Reverse 5′TCT GAC AGT TCG CAC AGG AC 3′

IL-4	Forward 5′ TGC-CGG-CAA-CTT-TGT-CCA-CG 3′
	Reverse 5′ TGG-TGG-CTG-TAG-AAC-TGC-CGG-A 3′

IL-10	Forward 5′ AGG-AGG-TGA-TGC-CCC-AAG-CTG-A 3′
	Reverse 5′ TTC-TTC-ACC-TGC-TCC-ACG-GCC-T 3′

**Table 2:** Medical and obstetrical background of women.

Background	Control mothers	GDM mothers
Previous gestational diabetes	—	1 (10%)
High blood pressure	—	1 (10%)
Preeclampsia	—	1 (10%)
Miscarriage	2 (20%)	3 (30%)
Recurrent spontaneous abortions	—	7 (70%)
Stillbirth	1 (10%)	2 (20%)

%: the percentage of the occurrence of previous obstetric complications; *n* = 10 control mothers; *n* = 10 gestational diabetes mellitus (GDM) mothers.

**Table 3:** Anthropometrical and biochemical parameters.

Parameters	Mothers		Babies	
	Control	GDM	Control	Macrosomic
Age	30.6 ± 3.47	32.3 ± 4.3	H1	H1
Weight (kg)^a,b^	63.4 ± 3.9	84.4 ± 2.4^∗^	3.29 ± 0.33	4.585 ± 0.46^∗^
Height (m)	1.65 0.04	1.67 ± 0.04	0.493 ± 0.011	0.51 ± 0.009
BMI (kg/m^2^)^c^	23.21 ± 1.27	30.25 ± 1.9^∗^	—	—
Cranial perimeter (cm)	—	—	32.9 ± 1.45	35.5 ± 1.78^∗^
Glycemia (g/L)	0.70 ± 0.23	1.26 ± 0.24^∗^	0.908 ± 0.26	0.78 ± 0.065
Insulinemia (*μ*U/mL)	3.59 ± 2.26	17.02 ± 5.63^∗^	2.73 ± 1.77	5.81 ± 4.4^∗^
HbA1C (%)	4.46 ± 0.18	5.27 ± 0.18	—	—
Triglycerides (g/L)	0.51 ± 0.19	1.47 ± 0.31^∗^	0.31 ± 0.13	0.55 ± 0.25
Total cholesterol (g/L)	0.64 ± 0.29	2.33 ± 0.68^∗^	0.51 ± 0.15	0.42 ± 0.13
HDL cholesterol (g/L)	0.39 ± 0.15	0.84 ± 0.19^∗^	0.30 ± 0.08	0.36 ± 0.10
LDL cholesterol (g/L)	0.15 ± 0.16	1.2 ± 0.52^∗^	0.061 ± 0.04	0.14 ± 0.08

^∗^Significant difference between diabetic mothers and macrosomic newborns and their corresponding controls; ^∗^*P* < 0.05. ^a^Mother's weight measured in the first trimester of pregnancy. ^b^Babies weight measured in the first hour after birth. ^c^Body mass index (BMI) of mothers, calculated with the following formula: BMI (kg/m^2^) = weight/(height)^2^. H1 = first hour after birth.

**Table 4:** Ratio of Th1/Th2 mRNA of transcription factors and cytokines.

Th1/Th2 ratio	Mothers		Newborns	
	Control	Diabetic	Control	Macrosomic
T-bet/GATA3	4.73	6.44^∗^	1.031	5.48^∗^
IL-2/IL-4	1.093	5.74^∗^	0.36	2.03^∗^
IL-2/IL-10	0.034	0.95^∗^	0.03	0.57^∗^
IFN-*γ*/IL-4	4.16	22.7^∗^	0.22	1.14^∗^
IFN-*γ*/IL-10	0.13	3.76^∗^	0.018	0.32^∗^

Relative quantity of mRNA in different groups was determined, and RQ values were used to calculate the Th1/Th2 mRNA ratio in mothers (control and gestational diabetic) and newborns (control and macrosomic). ^∗^Significant difference between diabetic mothers and macrosomic newborns as compared to their corresponding controls.

## Data Availability

The data used to support the findings of this study are available from the corresponding author upon request.

## Abbreviations

HbA1C: Glycated hemoglobin
TG: Triglyceride
Tchol: Total cholesterol
HDLchol: High-density lipoprotein cholesterol
LDLchol: Low-density lipoprotein cholesterol
RT-qPCR: Quantitative reverse transcription-polymerase chain reaction
T-bet: Trans-acting T cell-specific transcription factor T-bet
IL-2: Interleukin-2
IFN-*γ*: Interferon-*γ*
GATA3: Trans-acting T cell-specific transcription factor GATA3
IL-4: Interleukin-4
IL-10: Interleukin-10.

## References

[1] Buchanan T. A., Xiang A., Kjos S. L., Watanabe R. (2007). What is gestational diabetes?. *Diabetes Care*.

[2] American Diabetes Association (2004). Gestational diabetes mellitus. *Diabetes Care*.

[3] Hill J. C., Krishnaveni G. V., Annamma I., Leary S. D., Fall C. H. D. (2005). Glucose tolerance in pregnancy in South India: relationships to neonatal anthropometry. *Acta Obstetricia et Gynecologica Scandinavica*.

[4] England L. J., Dietz P. M., Njoroge T. (2009). Preventing type 2 diabetes: public health implications for women with a history of gestational diabetes mellitus. *American Journal of Obstetrics and Gynecology*.

[5] Yang H., Wei Y., Gao X. (2009). Risk factors for gestational diabetes mellitus in Chinese women: a prospective study of 16,286 pregnant women in China. *Diabetic Medicine*.

[6] Dunne F. P. (1999). Pregestational diabetes mellitus and pregnancy. *Trends in Endocrinology & Metabolism*.

[7] Kyne-Grzebalski D., Wood L., Marshall S. M., Taylor R. (1999). Episodic hyperglycaemia in pregnant women with well-controlled type 1 diabetes mellitus: a major potential factor underlying macrosomia. *Diabetic Medicine*.

[8] Lepercq J., Taupin P., Dubois-Laforgue D. (2001). Heterogeneity of fetal growth in type 1 diabetic pregnancy. *Diabetes & Metabolism*.

[9] Star J., Carpenter M. W. (1998). The effect of pregnancy on the natural history of diabetic retinopathy and nephropathy. *Clinics in Perinatology*.

[10] Dandona P., Aljada A., Bandyopadhyay A. (2004). Inflammation: the link between insulin resistance, obesity and diabetes. *Trends in Immunology*.

[11] Dandona P., Aljada A., Chaudhuri A., Mohanty P., Garg R. (2005). Metabolic syndrome: a comprehensive perspective based on interactions between obesity, diabetes, and inflammation. *Circulation*.

[12] Mothe-Satney I., Filloux C., Amghar H. (2012). Adipocytes secrete leukotrienes: contribution to obesity-associated inflammation and insulin resistance in mice. *Diabetes*.

[13] Khan N. A. (2006). Inflammation et immunité: implications dans l’obésité et le diabète de type 2. *Oléagineux, Corps gras, Lipides*.

[14] Nasta F., Ubaldi V., Pace L., Doria G., Pioli C. (2006). Cytotoxic T-lymphocyte antigen-4 inhibits GATA-3 but not T-bet mRNA expression during T helper cell differentiation. *Immunology*.

[15] Abbas A. K., Lichtman A. H., Pillai S., Masson P. L. (2016). *Les bases de l’immunologie fondamentale et clinique. Traduit de la 3éme édition anglaise*.

[16] Giordano C. (1990). Immunobiology of normal and diabetic pregnancy. *Immunology Today*.

[17] Hichami A., Grissa O., Mrizak I., Benammar C., Khan N. A. (2016). Role of T-cell polarization and inflammation and their modulation by n-3 fatty acids in gestational diabetes and macrosomia. *Journal of Nutrition and Metabolism*.

[18] Khan N., Yessoufou A., Kim M., Hichami A. (2006). N-3 fatty acids modulate Th1 and Th2 dichotomy in diabetic pregnancy and macrosomia. *Journal of Autoimmunity*.

[19] Yessoufou A., Hichami A., Besnard P., Moutairou K., Khan N. A. (2006). Peroxisome proliferator-activated receptor *α* deficiency increases the risk of maternal abortion and neonatal mortality in murine pregnancy with or without diabetes mellitus: modulation of T cell differentiation. *Endocrinology*.

[20] Atègbo J.-M., Grissa O., Yessoufou A. (2006). Modulation of adipokines and cytokines in gestational diabetes and macrosomia. *The Journal of Clinical Endocrinology & Metabolism*.

[21] Mrizak I., Grissa O., Henault B. (2014). Placental infiltration of inflammatory markers in gestational diabetic women. *General Physiology and Biophysics*.

[22] Triboulot C., Hichami A., Denys A., Khan N. A. (2001). Dietary (n-3) polyunsaturated fatty acids exert antihypertensive effects by modulating calcium signaling in T cells of rats. *The Journal of Nutrition*.

[23] Yessoufou A., Moutairou K. (2011). Maternal diabetes in pregnancy: early and long-term outcomes on the offspring and the concept of “metabolic memory”. *Experimental Diabetes Research*.

[24] Yessoufou A., Nekoua M. P., Gbankoto A., Mashalla Y., Moutairou K. (2015). Beneficial effects of omega-3 polyunsaturated fatty acids in gestational diabetes: consequences in macrosomia and adulthood obesity. *Journal of Diabetes Research*.

[25] Catalano P. M., Kirwan J. P., Haugel-de Mouzon S., King J. (2003). Gestational diabetes and insulin resistance: role in short- and long-term implications for mother and fetus. *The Journal of Nutrition*.

[26] Catalano P. M., Huston L., Amini S. B., Kalhan S. C. (1999). Longitudinal changes in glucose metabolism during pregnancy in obese women with normal glucose tolerance and gestational diabetes mellitus. *American Journal of Obstetrics and Gynecology*.

[27] Catalano P. M. (2014). Trying to understand gestational diabetes. *Diabetic Medicine*.

[28] Grissa O., Yessoufou A., Mrisak I. (2010). Growth factor concentrations and their placental mRNA expression are modulated in gestational diabetes mellitus: possible interactions with macrosomia. *BMC Pregnancy and Childbirth*.

[29] Pribylova H., Dvorakova L. (1996). Long-term prognosis of infants of diabetic mothers: relationship between metabolic disorders in newborns and adult offspring. *Acta Diabetologica*.

[30] Merzouk H., Madani S., Chabane Sari D., Prost J., Bouchenak M., Belleville J. (2000). Time course of changes in serum glucose, insulin, lipids and tissue lipase activities in macrosomic offspring of rats with streptozotocin-induced diabetes. *Clinical Science*.

[31] Comtois R., Séguin M. C., Aris-Jilwan N., Couturier M., Beauregard H. (1993). Comparison of obese and non-obese patients with gestational diabetes. *International Journal of Obesity and Related Metabolic Disorders*.

[32] Mayer G., Hudrisier D. Cytokines and Immunoregulation. *Immunology - chapter thirteen – Page maintained by Richard Hunt-last changed on Wednesday, November 08, 2017*.

[33] Jayabalan N., Nair S., Nuzhat Z. (2017). Cross talk between adipose tissue and placenta in obese and gestational diabetes mellitus pregnancies via exosomes. *Frontiers in Endocrinology*.

[34] Ingvorsen C., Brix S., Ozanne S. E., Hellgren L. I. (2015). The effect of maternal inflammation on foetal programming of metabolic disease. *Acta Physiologica*.

[35] Martin A. M., Berger H., Nisenbaum R. (2009). Abdominal visceral adiposity in the first trimester predicts glucose intolerance in later pregnancy. *Diabetes Care*.

[36] Raghupathy R. (2001). Pregnancy: success and failure within the Th1/Th2/Th3 paradigm. *Seminars in Immunology*.

[37] Reinhard G., Noll A., Schlebusch H., Mallmann P., Ruecker A. V. (1998). Shifts in the TH1/TH2 balance during human pregnancy correlate with apoptotic changes. *Biochemical and Biophysical Research Communications*.

[38] Feuerer M., Herrero L., Cipolletta D. (2009). Lean, but not obese, fat is enriched for a unique population of regulatory T cells that affect metabolic parameters. *Nature Medicine*.

[39] Deiuliis J., Shah Z., Shah N. (2011). Visceral adipose inflammation in obesity is associated with critical alterations in T regulatory cell numbers. *PLoS One*.

[40] Nekoua M. P., Fachinan R., Atchamou A. K. (2016). Modulation of immune cells and Th1/Th2 cytokines in insulin-treated type 2 diabetes mellitus. *African Health Sciences*.

[41] Jasper M. J., Tremellen K. P., Robertson S. A. (2006). Primary unexplained infertility is associated with reduced expression of the T-regulatory cell transcription factor Foxp3 in endometrial tissue. *Molecular Human Reproduction*.

